# Yamabushitake Mushroom (*Hericium erinaceus* (Bull.) Pers. 1797) Mycelium Improves Reproductive System Dysfunction in Male Rats Induced by Polystyrene Microplastics

**DOI:** 10.3390/ijms26125735

**Published:** 2025-06-15

**Authors:** Yi-Yuh Hwang, Sabri Sudirman, En-Yu Wei, Ruei-Feng Shiu, Zwe-Ling Kong, Deng-Fwu Hwang

**Affiliations:** 1 Department of Food Science, National Taiwan Ocean University, Keelung 20224, Taiwan; 2graceyy@gmail.com (Y.-Y.H.); 11032015@email.ntou.edu.tw (E.-Y.W.); 2 Department of Fisheries Product Technology, Faculty of Agriculture, Universitas Sriwijaya, Indralaya 30662, Indonesia; sabrisudirman@unsri.ac.id; 3 Institute of Marine Environment and Ecology, National Taiwan Ocean University, Keelung 20224, Taiwan; rfshiu@mail.ntou.edu.tw

**Keywords:** Erinacine A, *Hericium erinaceus*, microplastics, oxidative stress, reproductive system

## Abstract

The use of plastic products has increased, leading to higher levels of plastic pollution, and it is becoming a major public health concern. Health risks—especially those related to reproductive system dysfunction caused by polystyrene microplastics (PS-MPs)—are emerging issues that require urgent attention. This study aimed to investigate the effects of erinacine A-enriched *Hericium erinaceus* mycelium (HE) on high-fat-diet- and PS-MP-induced reproductive system dysfunction in male rats. Reproductive dysfunction was induced by administering a high-fat diet followed by exposure to PS-MPs for six weeks. The results showed that HE treatment significantly reduced nitric oxide levels and enhanced glutathione peroxidase activity. Furthermore, HE supplementation significantly downregulated pro-inflammatory cytokines such as interleukin (IL)-6 and IL-1β. Additionally, HE treatment significantly increased Kiss1 concentration, upregulated follicle-stimulating hormone and testosterone levels, reduced the area of the seminiferous tubule lumen, and prevented a reduction in epithelial thickness. HE treatment also significantly increased sperm count and reduced sperm abnormalities. Based on these findings, HE supplementation helps prevent reproductive system dysfunction by reducing oxidative stress and pro-inflammatory cytokines. Therefore, erinacine A-enriched *H. erinaceus* mycelium could be considered a potential food supplement or functional food ingredient for the treatment of reproductive or testicular dysfunction.

## 1. Introduction

As modern lifestyles evolve, the consumption of plastic products has increased, resulting in higher levels of plastic pollution. Plastic pollution, particularly in the form of microplastics, has become a significant environmental and public health issue [1]. Microplastics (MPs) are small fragments of plastic, ranging from 1 μm to 5 mm in size. Key sources of microplastics include polyethylene bags, packaging materials, cosmetics, plastic containers, electrical devices, glass, and various other products [2]. Humans can be exposed to microplastics through the consumption of contaminated water and food or by inhaling particles present in the air. Once ingested or inhaled, microplastics can lead to a range of harmful effects, including immune system disruptions, an increased risk of reproductive toxicity, digestive issues, liver toxicity, and cancer [3,4,5].

Polystyrene microplastics (PS-MPs) are a type of microplastic commonly found alongside other major plastics such as polypropylene, polyvinyl chloride, and polyethylene [6]. Animal studies have shown that PS-MPs induce the production of pro-inflammatory factors, including interleukins (IL)-6, IL-1β, and tumor necrosis factor (TNF)-α, leading to abnormalities in sperm quality in mice [2,7]. Polystyrene microplastics have also been linked to male reproductive system dysfunctions [4,8,9]. A previous study reported that PS-MPs decreased testosterone levels in mouse models [10,11]. Additionally, previous research has reported that PS-MPs cause disruptions in spermatogenesis, impair sperm quality, and trigger testicular tissue inflammation in mice [6,12]. Moreover, PS-MPs can induce oxidative stress, which further decreases sperm quality and disrupts the blood–testis barrier [13]. Consequently, the health risks, particularly reproductive damage associated with PS-MPs exposure, are emerging concerns that require urgent attention.

*Hericium erinaceus* (Bull.) Pers. 1797 is an edible mushroom that has been recognized as a traditional medicine, especially in the East Asian region. This mushroom has traditionally been used as both food and medicine in China, Japan, and Korea [14]. It is also known as Houtougu in China, Yamabushitake in Japan, and Norugungdengi in Korea. It belongs to the Hericiaceae family in the Basidiomycetes class [15,16]. This mushroom contains numerous bioactive metabolites, including high-molecular-weight substances such as polysaccharides and polyphenols, as well as low-molecular-weight substances such as erinacines and hericenones [17]. A previous study has reported that around 253 metabolites are known from *Hericium* species, including cyathane diterpenes, steroids, benzofurans, alkaloids, phenols, chromenes, pyrones, and other miscellaneous metabolites [18]. Erinacine A is a bioactive cyathane diterpenoid found in the *H. erinaceus* mycelia [19]. *Hericium erinaceus* extracts reduce oxidative stress and regulates inflammatory cytokines, such as inducible nitric oxide synthase (iNOS), interleukin (IL)-1β, IL-6, and tumor necrosis factor (TNF)-α, in both cell and animal models [20,21]. It also exhibits a protective effect against hydrogen peroxide-induced apoptosis [22]. Additionally, the diterpenoids erinacine A, B, and C in *H. erinaceus* mycelium have shown potential therapeutic benefits [23,24]. Moreover, erinacine A can pass through the blood–brain barrier in rats to support the development of *H. erinaceus* as a functional food for neurohealth improvement [25]. Based on these previous studies, we hypothesized that *H. erinaceus* mycelium could also improve reproductive system dysfunction in a male rat model. However, no such study has been reported in the literature. Therefore, this study aimed to investigate the protective effects of erinacine A-enriched *Hericium erinaceus* mycelium on reproductive system dysfunction induced by a high-fat diet and polystyrene microplastics in male rats. This study provides the first in vivo evidence of its protective role against PS-MP-induced male reproductive dysfunction. It also aimed to evaluate the effects of erinacine A-enriched *H. erinaceus* mycelium on oxidative stress, inflammatory cytokines, reproductive hormones, and sperm morphology and quality.

## 2. Results

### 2.1. Effects of Erinacine A-Enriched H. erinaceus Mycelium (HE) on the Organ Weights of the Rats

The effects of erinacine A-enriched *H. erinaceus* mycelium (HE) on the weight of the kidney, spleen, abdominal fat, epididymal fat, and testes in rats are shown in Table 1. No significant differences (*p* > 0.05) were observed among the groups after 6 weeks of HE treatment.

### 2.2. HE Supplementation Regulates Plasma Nitric Oxide Concentration and Glutathione Peroxidase Activity

**Figure 1:**
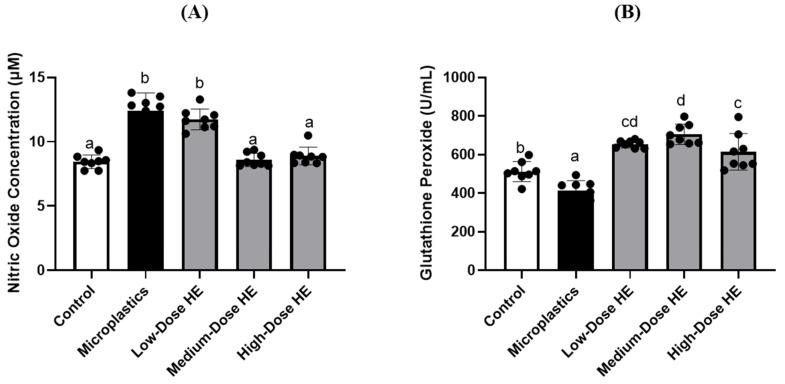
Effects of erinacine A-enriched *Hericium erinaceus* mycelium on (**A**) nitric oxide concentration and (**B**) glutathione peroxidase activity in plasma of rats after 6 weeks of treatment. Values with different letters (a–d) indicate significant differences (*p* < 0.05), as analyzed by Duncan’s multiple range test. Data are presented as mean ± SD (*n* = 8 rats/group). The circles (•) in each bar indicate individual data points; HE, Erinacine A-enriched *Hericium erinaceus* mycelium.

The nitric oxide (NO) level in the microplastics group was significantly increased (*p* < 0.05) compared to the control and treated groups (Figure 1). Conversely, glutathione peroxidase (GPx) activity was significantly decreased (*p* < 0.05) in the microplastics group compared to the control and treated groups. After 6 weeks of treatment with HE, NO levels were significantly reduced (*p* < 0.05), particularly with medium and high doses of HE, compared to the microplastics group. In contrast, GPx activity was significantly increased (*p* < 0.05).

### 2.3. HE Reduces the Levels of Interleukin-6 and Interleukin-1β in Rat Plasma

**Figure 2:**
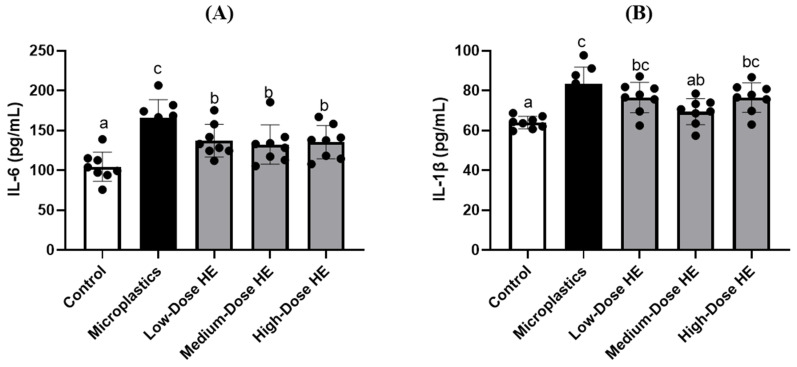
Effects of erinacine A-enriched *Hericium erinaceus* mycelium extract on (**A**) IL-6 and (**B**) IL-1β levels in plasma of rats after 6 weeks of treatment. Values with different letters (a–c) indicate significant differences (*p* < 0.05), as analyzed by Duncan’s multiple range test. Data are presented as mean ± SD (*n* = 8 rats/group). The circles (•) in each bar indicate individual data points; HE, Erinacine A-enriched *Hericium erinaceus* mycelium extract; IL, interleukin.

The interleukin (IL)-6 level significantly (*p* < 0.05) increased in the microplastics group compared to the control and treated groups (Figure 2). However, this level significantly (*p* < 0.05) decreased after treatment with different doses of HE for 6 weeks. As shown in Figure 2, the level of IL-1β also significantly (*p* < 0.05) increased in the microplastics group compared to the control group. However, treatment with the medium dose of HE significantly (*p* < 0.05) decreased the IL-1β level.

### 2.4. HE Regulates Kiss1 and Reproductive Hormone Concentrations

**Figure 3:**
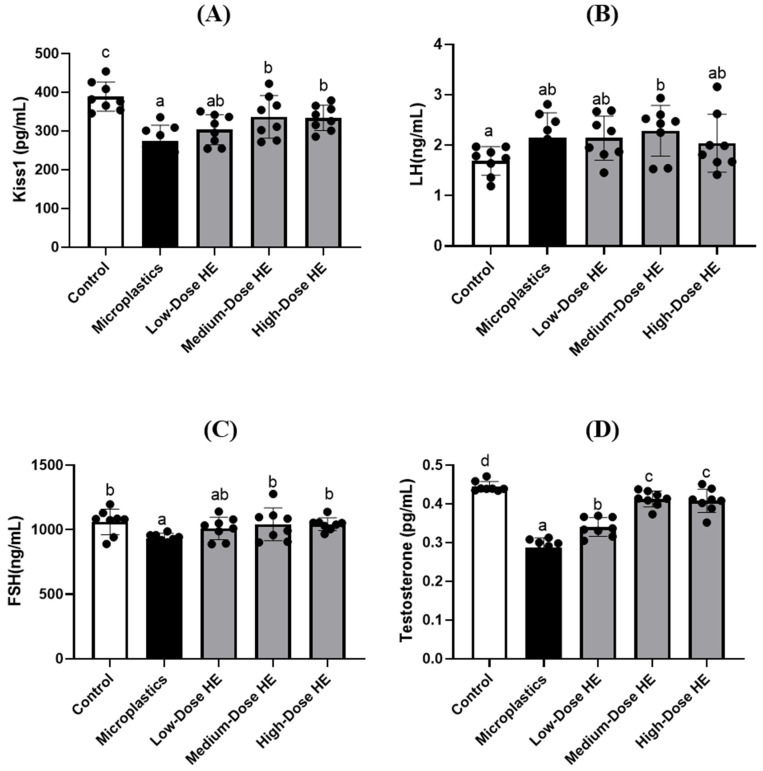
Effects of erinacine A-enriched *Hericium erinaceus* mycelium extract on (**A**) Kiss1 concentration, (**B**) LH, (**C**) FSH, and (**D**) testosterone hormones in plasma of rats after 6 weeks of treatment. Values with different letters (a–d) indicate significant differences (*p* < 0.05), as analyzed by Duncan’s multiple range test. Data are presented as mean ± SD (*n* = 8 rats/group). The circles (•) in each bar indicate individual data points; HE, Erinacine A-enriched *Hericium erinaceus* mycelium extract; LH, luteinizing hormone; FSH, follicle-stimulating hormone.

Kiss1 level was significantly (*p* < 0.05) reduced in the microplastics group compared to the control group, as shown in Figure 3. Treatment with HE for 6 weeks significantly (*p* < 0.05) increased Kiss1 levels, especially for the medium and high doses of HE. The reproductive hormones, such as luteinizing hormone (LH), follicle-stimulating hormone (FSH), and testosterone, were measured at the end of the experiment (Figure 3). FSH and testosterone levels were significantly different (*p* < 0.05) in the microplastics group compared to the control group. However, these levels significantly (*p* < 0.05) improved after treatment with medium and high doses of HE for 6 weeks. On the other hand, there were no significant differences (*p* > 0.05) in LH levels between the microplastics and control groups, as well as the treated groups.

### 2.5. Effects of HE on the Sperm Count, Abnormality, and Motility

**Figure 4:**
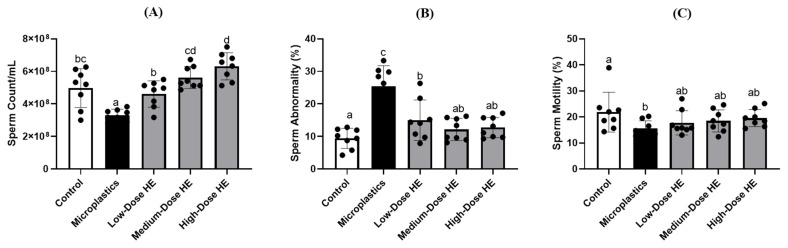
Effects of erinacine A-enriched *Hericium erinaceus* mycelium extract on the sperm (**A**) count, (**B**) abnormality, and (**C**) motility of the rats after 6 weeks of treatment. The values with different letters (a–d) represent significant difference (*p* < 0.05) as analyzed by Duncan’s multiple range test. Data are shown as mean ± SD (*n* = 8 rats/group). The circles (•) in each bar indicate individual data points; HE, Erinacine A-enriched *Hericium erinaceus* mycelium.

The rat sperm count in the microplastics group was significantly reduced (*p* < 0.05) compared to the control and treated groups (Figure 4). In contrast, sperm abnormalities were significantly increased (*p* < 0.05) in the microplastics group compared to the control and treated groups. These sperm properties were significantly (*p* < 0.05) improved after treatment with HE for 6 weeks. Additionally, there was no significant difference (*p* > 0.05) in sperm motility between the microplastics and treated groups.

### 2.6. Effects of the HE on the Seminiferous Tubule’s Lumen Area and Thickness of the Epithelium

**Figure 5:**
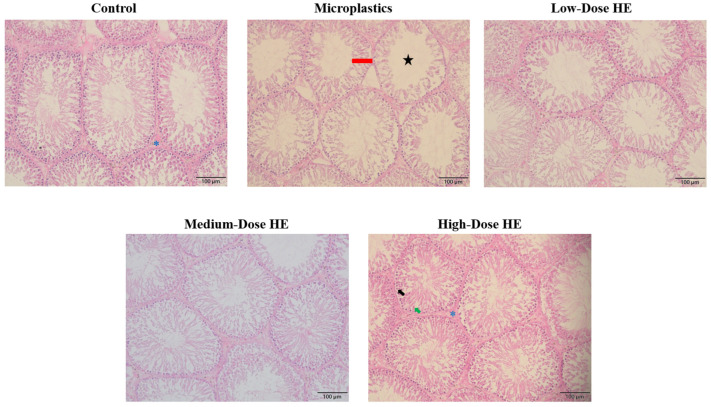
The representative morphology of seminiferous tubules in each rat group after 6 weeks of treatment with HE. HE, Erinacine A-enriched *Hericium erinaceus* mycelium. Figure legends: Blue asterisk, interstitial tissue containing Leydig cell; black star, seminiferous lumen; red bar, seminiferous epithelium; green arrow, Sertoli cell; black arrow, spermatogonia.

The morphology of the seminiferous tubule lumen and epithelium in rats after treatment with erinacine A-enriched *H. erinaceus* mycelium (HE) is shown in Figure 5, while the seminiferous tubules lumen area and epithelium thickness are presented in Table 2. These data indicate a significant (*p* < 0.05) increase in seminiferous tubule area in the microplastics group. Consequently, the epithelium thickness significantly (*p* < 0.05) decreased in this group. However, the values of the seminiferous tubule area and the thickness of the epithelium significantly (*p* < 0.05) improved after treatment with HE.

## 3. Discussion

This study presents the first in vivo evidence of the protective effects of erinacine A-enriched *Hericium erinaceus* mycelium against male reproductive dysfunction induced by polystyrene microplastics. However, it has some limitations, such as the absence of a positive control and a lack of primary data on the underlying molecular mechanisms, including no direct evidence of the modulation of specific molecular pathways.

In this present study, the microplastic-induced and erinacine A-enriched *H. erinaceus* mycelium (HE) had no effect on organ weights, including the kidney, spleen, abdominal fat, epididymal fat, and testis (Table 1). However, a reduction in glutathione peroxidase (GPx) activity was observed in the microplastics group, which resulted in an increase in nitric oxide (NO) levels in this group (Figure 1). A previous study also reported that polystyrene microplastics induce oxidative stress by increasing nitric oxide (NO) and malondialdehyde (MDA) levels, while reducing enzymatic antioxidant activity, including GPx [8]. Glutathione peroxidase is an enzymatic antioxidant that plays a role in regulating oxidative stress in the body. This enzyme converts and reduces hydrogen peroxide to water, thereby decreasing its adverse effects [26]. The GPx activity increased after treatment with HE for 6 weeks, accompanied by a decrease in nitric oxide (NO) concentration (Figure 1). A previous study reported that GPx catalyzes the breakdown of various oxidative species, including peroxynitrite (ONOO^−^) [27]. In a mouse model, erinacine A-enriched *H. erinaceus* mycelium also reduced iNOS levels and regulated oxidative stress [28]. The present study indicates that HE effectively decreases oxidative stress in the rat model. Additionally, erinacine A-enriched *H. erinaceus* mycelium enhanced the expression of antioxidant enzymes and Nrf2 nuclear proteins [19].

Figure 2 shows an increase in some pro-inflammatory cytokines, such as interleukin (IL)-6 and IL-1β, in the microplastics groups. A previous study also reported that microplastics significantly induced plasma IL-6 and tumor necrosis factor (TNF)-α in a rat model [8]. In a mouse study, polystyrene microplastics also elevated pro-inflammatory cytokines, such as TNF-α, IL-6, and IL-1β [7] The level of IL-6 was successfully reduced after treatment with various doses of HE. A previous study reported that erinacine A-enriched *H. erinaceus* mycelium also decreased IL-6 and TNF-α, pro-inflammatory cytokines [29]. This condition indicates that HE exhibits anti-inflammatory activity. We hypothesize that HE acts as an anti-inflammatory agent by inhibiting the translocation of the nuclear factor (NF)-κB transcription factor. As reported by a previous study, HE could block the phosphorylation of NF-κB and its translocation into the nucleus, resulting in a reduction in pro-inflammatory mediators [29,30].

The level of Kiss1 is reduced in the microplastic group (Figure 3). Kisspeptin is a neuropeptide encoded by the kisspeptin 1 (KISS1/Kiss1) gene. This peptide plays an important role in reproductive function, including in the release of reproductive hormones by stimulating gonadotropin-releasing hormone (GnRH) [31,32]. On the other hand, a decrease in some reproductive hormones, such as follicle-stimulating hormone (FSH) and testosterone, is observed in the microplastic group (Figure 3). This condition is due to the lower Kiss1 concentration observed in the present study. A previous study also reported that polystyrene microplastics reduce testosterone levels in a rat model [8]. In the present study, the level of Kiss1 increased after treatment with HE for 6 weeks, resulting in the improvement of some reproductive hormones, including FSH and testosterone.

The sperm count is reduced in the microplastics group, whereas sperm abnormalities are increased (Figure 4). This condition is due to the lower levels of reproductive hormones in this group. A previous study also reported that lower testosterone levels induced poor sperm quality [33]. Following improvements in FSH and testosterone levels, an increase in sperm count and a reduction in sperm abnormalities were observed in the treated group (Figure 4). A previous study reported that at intermediate and high levels of FSH, the total sperm count was successfully improved [34]. Testosterone is the testicular androgen necessary for the initiation and maintenance of spermatogenesis [35]. Therefore, the improvement of sperm abnormality is also observed after treatment with HE for 6 weeks. As reported by previous studies, testosterone regulates the sperm morphology [33,36]. We hypothesized that the improvement in sperm quality is also associated with the reduction in oxidative stress by HE. In the present study, HE successfully enhanced GPx activity and reduced oxidative stress. Oxidative stress, particularly in sperm, causes adverse effects by damaging sperm DNA and triggering apoptosis in sperm cells [37]. A previous study reported that increasing antioxidant activity protects tissues from oxidative stress, resulting in improvements in sperm count and function [38,39]. Additionally, erinacine A-enriched *H. erinaceus* mycelium exhibits free radical scavenging activity [40]. Together with the reduction in oxidative stress, HE also improves the sperm quality through its anti-inflammatory activity. A previous study reported that the negative effects on spermatogenesis are caused by oxidative stress, primarily resulting from elevated levels of ROS and pro-inflammatory cytokines [41].

Figure 5 shows the increase in the seminiferous tubules’ lumen area/space in the microplastics group, as well as the shrinkage of the seminiferous tubule epithelium. This condition indicates a low concentration of elongated spermatids and sperm cells. As reported by previous studies, the seminiferous tubule lumen is the site where developing sperm cells are located [42,43]. The morphology of the seminiferous tubules improved with treatment using HE for 6 weeks. This condition confirms that HE successfully protects the testis from the adverse effects of polystyrene microplastics.

In this study, erinacine A-enriched *H. erinaceus* mycelium successfully improved the reproductive system of the rats. This effect was due to the reduction in oxidative stress and certain pro-inflammatory cytokines by the erinacine A-enriched *H. erinaceus* mycelium. A previous study reported that oxidative stress and inflammation are important factors in the pathophysiology of infertility [44]. The pro-inflammatory cytokines and oxidative stress also led to a reduction in kisspeptin, resulting in decreased reproductive hormones [45]. Therefore, the inhibition of oxidative stress and pro-inflammatory cytokines by various doses of erinacine A-enriched *H. erinaceus* mycelium resulted in an improved reproductive system. A previous study reported that erinacine A-enriched H. erinaceus mycelium activates the BDNF/TrkB/PI3K/Akt/GSK-3β pathways and inhibits nuclear factor (NF)-κB transcription in an animal model [29]. Furthermore, NF-κB is a transcription factor that regulates the expression of pro-inflammatory cytokines [46].

## 4. Materials and Methods

### 4.1. Materials

Erinacine A-enriched *Hericium erinaceus* (Bull.) Pers. 1797 mycelium was provided by Grape King Biotechnology Co., Ltd. (Taoyuan City, Taiwan). The presence of erinacine A in the *H. erinaceus* mycelium has been confirmed according to previous studies [47,48]. The polystyrene microplastics (PS-MPs) were acquired from the Cell-Bio Biotechnology Co., Ltd. (New Taipei City, Taiwan) with a particle size ranging from 0.4 to 0.6 μm and a concentration of 10% (*w*/*v*) (Cat. No. DNM-P004). Roswell Park Memorial Institute (RPMI) medium, interleukin (IL)-1β (Cat. No. BMS630), IL-6 (Cat. No. ERA31RB), and tumor necrosis factor (TNF)-α (Cat. No. KRC3011) ELISA kits were purchased from Thermo Fisher Scientific (Waltham, MA, USA). Glutathione peroxidase (GPx) commercial kit was purchased from Randox Laboratories (Crumlin, County Antrim, UK). Luteinizing hormone (LH, Cat. No. ER1123), follicle-stimulating hormone (FSH, Cat. No. ER0960), testosterone (Cat. No. ER1462), and Kiss1 (Cat. No. ER0628) ELISA kits were purchased from Wuhan Fine Biotech (Wuhan, China).

### 4.2. Polystyrene Microplastics Solution Preparation

A 10% (*w*/*v*) solution of polystyrene microplastics (PS-MPs) was prepared in deionized water and treated with ultrasonic vibration for 30 min to ensure uniform dispersion of the plastic particles. Fresh suspensions were prepared daily to maintain consistency and prevent aggregation [49].

### 4.3. Animal Study

#### 4.3.1. Animal Treatment

**Figure 6:**
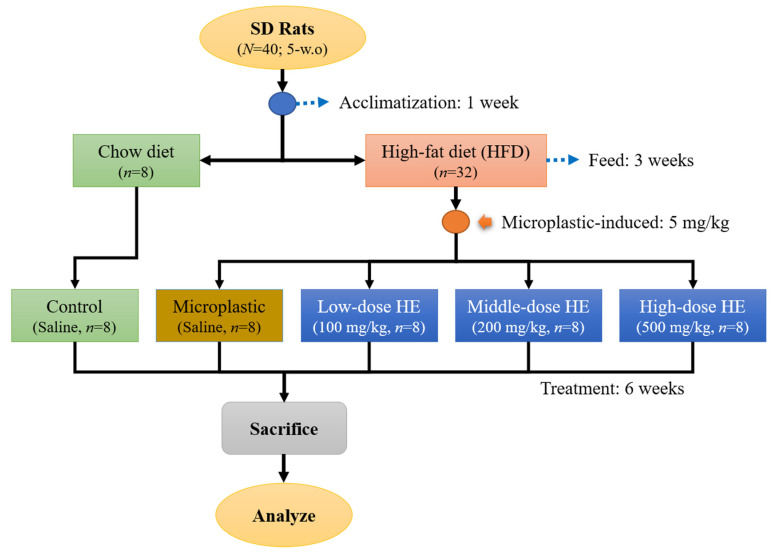
Flowchart of high-fat-diet- and microplastic-induced rats treated with *Hericium erinaceus* mycelium (HE) for 6 weeks. HE, Erinacine A-enriched *H. erinaceus* mycelium; HFD, high-fat diet; SD, Sprague Dawley.

Forty male Sprague Dawley (SD) rats (5 weeks old, *N* = 40)) were housed individually and provided with water ad libitum and a standard chow diet (LabDiet 5001). The rats were maintained under standard laboratory conditions (temperature 23 ± 1 °C, humidity 40–60%, 12-h light/dark cycle). The rats were acclimatized for a week. The Institutional Animal Care and Use Committee (IACUC Approval No. 111046, Date: 1 August 2023) of the National Taiwan Ocean University reviewed and approved all protocols. After the acclimatization phase, the rats were randomly divided into two main groups (Figure 6). A group continued to be fed by chow diet (control, *n* = 8) and another group was fed with a high-fat diet (HFD) for 3 weeks (*n* = 32). After that, the HFD group was randomly divided into 4 groups. These group were daily induced with 5 mg/kg (oral gavage) polystyrene microplastics (PS-MPs) and the dose of PS-MPs was adapted from a previous study [8]. Then, these groups were either treated with various doses of erinacine A-enriched *H. erinaceus* mycelium (HE) or none; a group without HE treatment (Microplastic group, *n* = 8) and three groups were either treated by daily oral gavage with 100 mg/kg body weight of HE (Low-dose HE, *n* = 8), 200 mg/kg (Medium-dose HE, *n* = 8), or 500 mg/kg (High-dose HE, *n* = 8). The doses used were adapted from previously published methods [50,51]. The control and microplastic groups were administered daily with a saline solution. The rats were sacrificed after treatment for 6 weeks. The rats fasted prior to sacrifice for 12 h. They were sacrificed in an empty chamber through exposure to CO_2_ until euthanasia [52]. Blood was collected for further analysis and the organs (kidney, spleen, testes) as well as abdominal and epididymis fat were weighed at the day of sacrifice. One of the testes was soaked in 4% formaldehyde solution for histopathological analysis.

#### 4.3.2. Blood Sample Collection

The blood was collected from the rats on the day of sacrifice. The blood was collected from the rats’ abdominal aorta. Then, it was centrifuged at 3000 rpm for 15 min at 4 C to separate the plasma. The plasma (supernatant) was collected and stored at freezing temperatures (−25 °C) for subsequent analysis [53].

#### 4.3.3. Nitric Oxide and Glutathione Peroxidase Analysis

The nitric oxide (NO) content was determined using the Griess reagent, which consists of 1% (*w*/*v*) sulphanilamide in 5% (*v*/*v*) phosphoric acid and 0.1% (*w*/*v*) N-(1-naphthyl) ethylenediamine-HCl, following a previously described method [54]. In brief, 100 μL of rat plasma from each group and 100 μL of serially diluted standards were transferred to a new 96-well plate, then mixed with an equal volume of Griess reagent and incubated for 10 min. Absorbance was measured at 540 nm using a microplate spectrophotometer. Plasma enzymatic antioxidant activities, such as glutathione peroxidase (GPx), were analyzed according to the manufacturer’s protocol for the Randox Laboratories kit.

#### 4.3.4. Pro-Inflammatory Cytokines, Kiss1, and Reproductive Hormones Analysis

The plasma pro-inflammatory cytokines, such as interleukin (IL)-6 and IL-1β, as well as Kiss1 concentration and reproductive hormones such as luteinizing hormone (LH), follicle-stimulating hormone (FSH), and testosterone concentrations, were analyzed using enzyme-linked immunosorbent assay (ELISA) kits performed according to the manufacturer’s protocols.

#### 4.3.5. Sperm Count, Abnormality, and Motility Analysis

The swim-up method was used to collect sperm from the epididymis. Epididymis was cut into 3 pieces, immersed in 8 mL Roswell Park Memorial Institute (RPMI) medium and shaken in an orbital shaker at 100 rpm for 10 min. Epididymis with RPMI medium was centrifuged at 100× *g* for 5 min and incubated at 37 °C in a 5% CO_2_ incubator for 30 min. Finally, the sperms were collected from the supernatant and observed under the microscope to determine the sperm count, abnormality, and motility [55].

#### 4.3.6. Testis Histopathology Analysis

The testis was cut into 5 mm sections from the middle part using a scalpel. The group was marked on the lid and then it was soaked in a 4% formaldehyde solution at room temperature for paraffin embedding and hematoxylin and eosin (H&E) staining. The staining was performed by Rapid Science Co., Ltd. (Taichung, Taiwan). The testicular tissue section images were used ImageJ (ver. 1.54p) microscope image processing software to determine the seminiferous tubules’ lumen area and thickness of the epithelium to evaluate the impact on seminiferous tubules by polystyrene microplastics [56].

### 4.4. Statistical Analysis

The data were presented as mean ± standard deviation (SD) and analyzed by one-way analysis of variance (one-way ANOVA) followed by Duncan’s multiple comparison tests; *p* < 0.05 was considered significantly different. Statistical analysis was performed with Statistical Product and Service Solution (SPSS) 22.0 software (IBM Corporation, Armonk, NY, USA).

## 5. Conclusions

Polystyrene microplastics increase oxidative stress and pro-inflammatory cytokines, while also reducing sperm quality and altering seminiferous tubule morphology. However, treatment with erinacine A-enriched *Hericium erinaceus* mycelium effectively enhances glutathione peroxidase activity, reduces pro-inflammatory cytokines, and increases Kiss1, follicle-stimulating hormone, and testosterone levels. These improvements lead to enhanced sperm quality and seminiferous tubule structure. Overall, erinacine A-enriched *H. erinaceus* mycelium exerts protective effects against reproductive dysfunction by regulating oxidative stress and inflammatory cytokine levels. Therefore, it holds potential as a source of food supplements or functional food ingredients for the treatment of reproductive system dysfunctions. However, further investigation is needed to assess its bioavailability, stability, and consumer acceptance.

## Figures and Tables

**Table 1:** Effect of erinacine A-enriched Hericium erinaceus mycelium (HE) on organ weights (% of body weight) in rats.

Group	Kidney	Spleen	Abdominal Fat	Testes	Epididymis Fat
Control	4.23 ± 0.35	0.83 ± 0.18	8.83 ± 1.26	3.89 ± 0.89	8.02 ± 1.26
Microplastics	3.98 ± 0.37	0.74 ± 0.08	7.18 ± 1.98	3.74 ± 0.78	6.41 ± 1.75
Low-Dose HE	4.08 ± 0.48	0.70 ± 0.10	8.76 ± 2.45	4.43 ± 1.38	7.07 ± 2.24
Medium-Dose HE	4.26 ± 0.27	0.74 ± 0.10	7.93 ± 2.75	3.84 ± 0.93	7.40 ± 2.16
High-Dose HE	4.08 ± 0.55	0.76 ± 0.17	9.17 ± 2.34	3.79 ± 0.84	6.82 ± 2.29

Data are shown as the mean ± SD (*n* = 8 rats/group). HE, erinacine A-enriched Hericium erinaceus mycelium.

**Table 2:** Effects of erinacine A-enriched Hericium erinaceus mycelium extract on the seminiferous tubule lumen area and epithelium thickness in rats after 6 weeks of treatment.

Groups	Seminiferous Tubule Area (%)	Thickness of Epithelium (µm)
Control	17.55 ± 2.22 ^a^	110.23 ± 31.89 ^b^
Microplastics	41.74 ± 8.54 ^b^	62.03 ± 9.19 ^a^
Low-Dose HE	20.77 ± 8.03 ^a^	91.91 ± 13.72 ^b^
Medium-Dose HE	12.86 ± 0.61 ^a^	104.25 ± 13.93 ^b^
High-Dose HE	13.14 ± 1.57 ^a^	109.64 ± 14.06 ^b^

The values with different letters (a,b) in a column represent significant difference (*p* < 0.05) as analyzed by Duncan’s multiple range test. Data are shown as the mean ± SD (*n* = 8 rats/group). HE, Erinacine A-enriched Hericium erinaceus mycelium.

## Data Availability

The original contributions presented in this study are included in the article. Further inquiries can be directed to the corresponding author.

## Abbreviations

The following abbreviations are used in this manuscript:

ELISA	Enzyme-linked immunosorbent assays
FSH	Follicle-stimulating hormone
GPx	Glutathione peroxidase
HE	Erinacine A-enriched *H. erinaceus* mycelium
IL	Interleukin
LH	Luteinizing hormone
MDA	Malondialdehyde
NO	Nitric oxide
PS-MPs	Polystyrene microplastics
ROS	Reactive oxygen species
RPMI	Roswell Park Memorial Institute
TNF	Tumor necrosis factor
